# Research progress on resistance exercise therapy for improving cognitive function in patients with AD and muscle atrophy

**DOI:** 10.3389/fnagi.2025.1552905

**Published:** 2025-04-08

**Authors:** Wenyao Li, Wei Fang, Yier Zhang, Qiulu Chen, Wuyue Shentu, Qilun Lai, Lin Cheng, Sicheng Yan, Qi Kong, Song Qiao

**Affiliations:** ^1^Department of Special Inspection, Hangzhou TCM Hospital Affiliated to Zhejiang Chinese Medical University, Hangzhou, China; ^2^Department of Neurology, Hangzhou TCM Hospital Affiliated to Zhejiang Chinese Medical University, Hangzhou, China; ^3^Zhejiang Chinese Medical University Hangzhou, Hangzhou, Zhejiang, China; ^4^Department of Neurology, Zhejiang Hospital, Hangzhou, Zhejiang, China; ^5^Department of Neurology, Zhejiang Medical and Health Group Hangzhou Hospital, Hangzhou, Zhejiang, China; ^6^Liuzhou People's Hospital, Liuzhou, Guangxi, China

**Keywords:** Alzheimer's disease, cognitive function, skeletal muscle atrophy, resistance exercise, neuromuscular

## Abstract

Alzheimer's disease (AD) significantly reduces the quality of life of patients and exacerbates the burden on their families and society. Resistance exercise significantly enhances the overall cognitive function of the elderly and patients with AD while positively improving memory, executive function, and muscle strength, reducing fall risks, and alleviating psychological symptoms. As AD is a neurodegenerative disorder, some nerve factors are readily activated and released during exercise. Therefore, several prior studies have concentrated on exploring the molecular mechanisms of resistance exercise and their impact on brain function and neural plasticity. Recent investigations have identified an intrinsic relationship between individuals with AD and the pathological mechanisms of skeletal muscle atrophy, establishing a correlation between patients with AD cognitive level and skeletal muscle content. Resistance exercise primarily targets the skeletal muscle, which improves cognitive impairment in patients with AD by reducing vascular and neuroinflammatory factors and further enhances cognitive function in patients with AD by restoring the structural function of skeletal muscle. Furthermore, the effects of resistance training vary among distinct subgroups of cognitive impairment. Individuals exhibiting lower cognitive function demonstrate more pronounced adaptive responses in physical performance over time. Consequently, further investigation is warranted to determine whether tailored guidelines—such as variations in the frequency and duration of resistance exercise—should be established for patients with varying levels of dementia, in order to optimize the benefits for those experiencing cognitive impairment. This study aimed to review the relationship between AD and skeletal muscle atrophy, the impact of skeletal muscle atrophy on AD cognition, the mechanism by which resistance exercise improves cognition through skeletal muscle improvement, and the optimal resistance exercise mode to elucidate the additional advantages of resistance exercise in treating cognitive function in patients with AD and skeletal muscle atrophy.

## 1 Introduction

Recently, with the aggravation of population aging, social development, the incidence rate of Alzheimer's disease (AD) has increased significantly, placing a substantial burden on families and society. The latest released statistics by the International AD Association indicate that the global prevalence of dementia is projected to reach 75 million by 2030 (Stephan et al., 2018). Therefore, AD research has gradually become a prominent subject of interest. Despite the ability of pharmacological interventions to improve cognitive function and behavioral symptoms in patients with AD, most patients do not receive effective treatment because of the large patient population and slow progress in drug development. The pertinent literature suggests that approximately 40% of global cognitive dysfunction is caused by 12 controllable risk factors, and AD constitutes over 60% of all cognitive dysfunction (Stephan et al., 2018). Consequently, patients, families, and society need to reduce the incidence of AD and delay cognitive decline by changing living environments, daily living habits, and other controllable risk factors. Current guidelines recommend resistance exercise as a non-pharmacological preventive intervention for cognitive impairment (Bangsbo et al., 2019). Although certain previously conducted randomized controlled trials (RCTs) have indicated that resistance training can improve and delay the behavior and cognition of AD, most trials have concentrated on the effects of resistance exercise on brain structure and function, including cerebrovascular function and cerebral blood flow perfusion, brain structure, synaptic development, and neurotrophic factors, to elucidate the mechanism by which resistance exercise enhances cognitive function in patients with AD (Ben-Zeev et al., 2022). Skeletal muscle atrophy can affect the cognition of patients with AD (Liu et al., 2023), and resistance exercise can improve the physiological structure and function of the skeletal muscles (Rahmati et al., 2023). Therefore, resistance exercise may directly improve skeletal muscle function and further enhance the cognitive function of patients with AD. Furthermore, there is a lack of recent research examining the physical and cognitive effects of resistance exercise on individuals with varying levels of cognitive impairment, indicating a need for further investigation. At the same time, there are multiple treatment options available for AD, such as resistance exercise, medication, cognitive training, yoga, Baduanjin, and other mind-body therapies. However, studies on the effectiveness of combining these treatments, particularly for patients with different levels of cognitive impairment, are scarce. Future research is essential to develop targeted and personalized treatment approaches.

## 2 The relationship between AD and skeletal muscle atrophy

AD is a neurodegenerative disease characterized by the progressive deterioration of cognitive behavior and ability (Jack et al., 2010). The characteristic pathological features of AD are the accumulation of extracellular amyloid plaques and intracellular neurofibrillary tangles in the brain (Querfurth and LaFerla, 2010). Starch-like protein plaques primarily comprise amyloid beta (Aβ) peptides, whereas over-phosphorylated tau is the principal component of intracellular neurofibrillary tangles. AD presents two manifestations: Early familial and late sporadic. The early familial type has a low incidence rate, accounting for only 1–2%. The premature-onset familial type is generally associated with amyloid precursor protein (APP), premature aging protein 1 (PS1 or PSEN1), and premature aging protein 2 mutations at the (PS2 or PSEN2) site, which are associated with excessive production of Aβ. Late sporadic type onset is a prevalent form of AD in patients aged 65 years or older exhibiting the APOE4 genotype. Changes in lifestyle, genetics, and environmental factors significantly affect the onset of the late sporadic type. As a result, current research has focused on the late sporadic type of AD.

### 2.1 AD impacts skeletal muscle atrophy

Several risk factors have been identified for the late sporadic type of AD, with aging being the principal cause (Robinson et al., 2023). Aging increases the risk of skeletal muscle atrophy. Although Aβ accumulation in the brain is primarily associated with AD, Aβ deposition and APP have been detected in the skeletal muscles of humans and certain animal models. Progressive loss of skeletal muscle function, including decreased muscle mass and strength, can also be observed in patients with AD (Ogawa et al., 2018; Burns et al., 2010; Fukuchi et al., 1998). Previous studies (Burns et al., 2010) have demonstrated a positive correlation between progressive cerebral atrophy and muscle mass reduction in patients with AD. Since 1984, abnormal weight loss and cachexia have been considered the clinical manifestations of AD. Previous studies (Sugimoto et al., 2016) have indicated that the risk of muscle loss in patients with AD is higher than in a population without cognitive impairment in the same age group. A study using magnetic resonance imaging (MRI) and dual-energy X-ray absorptiometry (DEXA) revealed that compared with the normal control group, patients with AD experienced significant weight loss, accompanied by cognitive decline and reduced brain volume (Burns et al., 2010). A subsequent study confirmed using an AD transgenic mouse model (3xTgAD mice) that compared to young (2–4 months) mouse models, elderly (18–20 months) mouse with AD exhibited better performance, including more phenotypes related to muscle atrophy, including neuromuscular junction injury, reduced gastrocnemius muscle mass, sciatic nerve induction, and direct muscle stimulation to decrease the induced contraction force (Xu et al., 2022). Moreover, Aβ levels were elevated in the skeletal muscle and neurogenic groups of elderly 3xTgAD mice, and the TGF-β-mediated atrophy signaling pathway was activated in elderly 3xTgAD mice, potentially contributing to muscle atrophy in this group. This study suggests that the pathological mechanism of AD involves peripheral alterations in the skeletal muscle. A prospective study elucidated the relationship between muscle loss and the AD continuum (Kim et al., 2024), which includes preclinical AD, mild cognitive impairment (MCI) caused by AD, and AD dementia (Sperling et al., 2011; Albert et al., 2011; McKhann et al., 2011). A total of 142 participants with AD continuum and 58 Aβ-negative cognitively normal patients were evaluated using DEXA and grip strength measurements. This study discovered an independent association between muscle loss and AD continuum. The skeletal muscles of patients with AD may be more susceptible to oxidative and inflammatory stress (Monteiro-Cardoso et al., 2015).

### 2.2 Skeletal muscle atrophy affects AD cognition

Numerous investigations have revealed that elderly individuals with Aβ in their brains exhibit minimal or no expression (Aizenstein et al., 2008; Johnson et al., 2013; Roberts et al., 2018). Consequently, in addition to cognitive impairment caused by Aβ, other factors may be linked to the deterioration of brain function. In elderly patients or patients with dementia, muscle atrophy and cognitive decline occur almost simultaneously. Skeletal muscle atrophy may also exacerbate the severity of cognitive impairment or accelerate its progression of cognitive impairment in patients with AD (Brisendine et al., 2024). Certain researchers (Brisendine et al., 2024) have identified that neuromuscular dysfunction occurs before cognitive dysfunction in a Transgenic mice with five familial AD (5xFAD) mouse model. Accordingly, researchers hypothesize that neural conduction in skeletal muscles is a precursor to significant cognitive dysfunction, indicating that muscle structure and function alterations may influence cognitive function. This aligns with a human study (Qian et al., 2022) that identified a positive correlation between decreased peripheral motor nerve conduction velocity and cognitive impairment in patients diagnosed with MCI and AD compared with a control group without cognitive impairment. Epidemiological evidence suggests a bidirectional relationship between musculoskeletal health and the occurrence of AD (Sui et al., 2022).

However, due to the predominance of cross-sectional studies, accurately establishing the causal relationship between skeletal muscle atrophy and AD cognitive impairment remains challenging. Future longitudinal studies are essential to elucidate the relationship between the two, facilitating more targeted interventions for the disease and focusing on improving skeletal muscle atrophy or alleviating AD cognition to maximize patient outcomes.

## 3 Possible mechanisms of skeletal muscle atrophy affecting cognitive function in AD

In recent years, the concept of a bidirectional relationship between bones and the brain, known as the bone-brain axis (Zhang and Zhang, 2024), has gained attention and this theory (Brazill et al., 2019; Millar et al., 2019) suggests that the brain not only influences bone health (efferent pathway) but that skeletal muscles can also send signals to the brain by releasing bone-derived factors (efferent pathway). These molecules may have effects on brain function, and studies have identified their presence in the brain (Brazill et al., 2019; Millar et al., 2019). An animal study (Kim et al., 2024) demonstrated a correlation between skeletal muscle atrophy and decreased hippocampal volume and cognitive function in patients with AD continuum. Skeletal muscle atrophy impacts cognitive abilities in AD, likely due to the interplay of several systems, primarily consisting of Oudbier et al. (2022): (1) systemic inflammation including decreased neurotrophic factors and myokines; (2) insulin irregularities; (3) disruptions in protein metabolism; (4) compromised mitochondrial function; (5) Others. These interconnected pathological processes collectively establish the biological foundation for the degenerative alterations in the bone-brain network.

### 3.1 Systemic inflammation

#### 3.1.1 Role of proinflammatory cytokines

In generally (Ramsey et al., 2021), older adults tend to be less physically active and have a greater presence of pro-inflammatory cells in their bodies. As a result, their muscle strength is often diminished compared to those who are more active. Increased levels of inflammatory markers in the bloodstream, like C-reactive protein and IL-6, have been linked to dementia (Darweesh et al., 2018). When released by type I and type II skeletal muscle fibers during muscle contraction, IL-6 acts as an anti-inflammatory cytokine (Pedersen and Febbraio, 2005). Research indicates (Schumertl et al., 2022) that IL-6 is crucial for maintaining balance in the central nervous system within the brain.

#### 3.1.2 Role of myokine

Research indicates that metabolically active tissues, including skeletal muscle, release neurotrophic factors for brain synapses, one of which is brain-derived neurotrophic factor (BDNF; Lu et al., 2014). BDNF is released during skeletal muscle contraction and is a neurotrophic factor required for maintaining synaptic connections and adaptive neuronal plasticity in adults. It can regulate cognitive processes, including learning and memory; its deficiency is associated with neurodegenerative processes (Lu et al., 2014).

Irisin is a newly identified muscle factor that is released during exercise. It is produced from the precursor protein fibronectin type III domain protein 5 (FNDC5) when skeletal muscles contract, and then it is cleaved and enters the bloodstream (Boström et al., 2012). In mice, 72% of irisin originates from skeletal muscle, while 28% comes from fat (Ruan et al., 2019; Shirvani and Rahmati-Ahmadabad, 2019). Irisin has the ability to influence various cellular signaling pathways across different organs, it is secreted by skeletal muscles during physical activity and can cross the blood-brain barrier (Ruan et al., 2019; Shirvani and Rahmati-Ahmadabad, 2019). A study (Sanesi et al., 2023) examining the effects of FNDC5/irisin on muscle atrophy revealed that after 4 weeks of muscle atrophy, FNDC5 levels and serum irisin concentrations decreased. However, treatment with recombinant irisin was able to reverse muscle atrophy. The primary mechanism appears to involve irisin's ability to directly inhibit muscle protein degradation and promote myosin synthesis, as well as its role in maintaining bone health by regulating the balance of osteoprotegerin (OPG) and receptor activator of nuclear factor kappa-B ligand (RANKL) and the apoptosis pathway. Recent research has highlighted the importance of FNDC5/irisin in providing neuroprotection in AD. In a recent study (Kim et al., 2025) using a three-dimensional cell culture model of AD, it was found that irisin can reduce A β protein levels, suggesting that it mitigates Aβ pathology by enhancing the activity and levels of neprilysin (NEP) secreted from astrocytes.

Research indicates that certain bone-derived factors, such as osteocalcin (Jaberi and Fahnestock, 2023), lipocalin 2 (LCN2; Mosialou et al., 2017), sclerostin (Shi et al., 2024), and Dickkopf-related protein (Dkk; Sato et al., 2024), can cross the blood-brain barrier via the bloodstream. Additionally, extracellular vesicles, which are secreted by nearly all cell types, play a vital role in cell communication and the exchange of biological information (Couch et al., 2021). The transmission of information through these vesicles is linked to various diseases (Faraldi et al., 2021). Evidence suggests that extracellular vesicles from skeletal muscles can be taken up by different organs, including the brain (Aswad et al., 2014). Studies (Aswad et al., 2014; Rodríguez and Cabello-Verrugio, 2024) have shown that when skeletal muscles with nerve connections experience injury, there are significant alterations in the miRNA profiles of the extracellular vesicles they release. These changes can significantly affect brain functions related to neuroplasticity, memory, sleep, and emotions (Delezie and Handschin, 2018). This points to the existence of pathways mediated by extracellular vesicles between the brain and bones, although more research is needed in this area.

### 3.2 Insulin metabolism

Skeletal muscle is crucial for regulating blood glucose levels, serving as a key organ for glucose storage and metabolism (Sylow et al., 2021). Muscle atrophy is linked to insulin resistance (Kim and Park, 2018), a significant risk factor for cognitive decline (Ekblad et al., 2017). A sustained rise in insulin levels in the body is associated with lower insulin levels in the brain, which decreases the clearance of Aβ (Cholerton et al., 2013). Another hypothesis suggests that elevated insulin levels in the body compete with enzymes that break down insulin, resulting in the buildup of A β and impaired degradation, which may contribute to increased tau formation (Nguyen et al., 2020).

### 3.3 Protein metabolism

The decline in skeletal muscle mass is caused by a reduction in muscle protein synthesis and an increase in muscle protein breakdown, resulting in a negative net protein balance (Kim et al., 2020). This negative balance can also lower protein levels in the brain, which can indirectly impact cognitive function. Furthermore, studies (Poddar et al., 2019) have shown that not only does protein content decrease, but the extent of oxidative damage to proteins also rises, contributing to cognitive impairment. Additionally, skeletal muscle atrophy is linked to the upregulation of the ubiquitin-proteasome system (UPS; Al Mamun et al., 2020), which is a system that promotes protein breakdown. The amyloid precursor protein (APP), associated with AD, has been confirmed to be related to the UPS (Al Mamun et al., 2020).

### 3.4 Mitochondrial function

The primary energy source for skeletal muscle contraction is adenosine triphosphate (ATP) (Gan et al., 2018), which is largely produced through mitochondrial oxidative phosphorylation. Mitochondrial issues, such as changes in quantity, function, and structure, are frequently observed in atrophied skeletal muscle (Picca et al., 2018). Additionally, mitochondrial dysfunction in the brain may contribute to cognitive decline. When skeletal muscle atrophy occurs, it can lead to mitochondrial dysfunction, resulting in the buildup of reactive oxygen species (ROS). An overproduction of ROS heightens oxidative stress, which in turn increases the production of Aβ (Leuner et al., 2012). Furthermore, it is understood that oxidative stress serves as a common underlying mechanism for both skeletal muscle atrophy and cognitive impairment (Liguori et al., 2018).

### 3.5 Others

Recent clinical studies have found that atrophied skeletal muscle releases hemoglobin, which can lead to cognitive dysfunction.

Elevated hemoglobin levels are linked to the progression of AD and are inversely related to cognitive function (Ashraf et al., 2020). In 2024, Iki and Tohda (2024) conducted experiments using both 5XFAD and non-transgenic wild-type mice, inducing skeletal muscle atrophy by immobilizing their hind limbs for 14 days. They found that continuous infusion of recombinant hemoglobin into the ventricles impaired object recognition memory in the 5XFAD mice with simulated muscle atrophy. Proteomic analysis showed that hemoglobin levels in the skeletal muscle, plasma, and hippocampus of these mice were higher than in typical 5XFAD mice. Furthermore, injecting hemoglobin into 5XFAD mice led to increased levels of lipocalin-2 (Lcn2), messenger RNA (mRNA), and neuroinflammatory markers in the hippocampus. The rise in LCN2 mRNA levels in the hippocampus of the simulated muscle atrophy mice indicates that skeletal muscle atrophy negatively impacts memory impairment in young 5XFAD mice, mediated by hemoglobin secretion from atrophied muscle. Thus, hemoglobin could be a potential therapeutic target for preventing cognitive decline in AD patients with skeletal muscle atrophy.

There is substantial research indicating that the factors mentioned earlier contribute to skeletal muscle atrophy and cognitive decline. However, it remains uncertain whether these mechanisms are exclusively a result of muscle atrophy or if there is a reciprocal relationship between muscle atrophy and cognitive impairment. Further investigation is required to clarify this issue in the future.

The atrophy and muscle loss of skeletal muscles are associated with chronic neurodegeneration and oxidative stress (Migliavacca et al., 2019), exacerbating the Aβ-related neurodegeneration process (Maltais et al., 2019). More longitudinal studies are needed to determine the relationship between the two, especially the relationship between muscle atrophy and Aβ deposition, which may be more convincing for the relationship between skeletal muscle atrophy and cognitive changes in AD. Since skeletal muscle functions as an endocrine organ capable of releasing various muscle factors, peptides, and growth factors (García-Llorente et al., 2024; Nicola et al., 2024), further research is necessary to investigate the pathways and molecular mechanisms involved in their signaling to the brain. Additionally, this research should aim to identify new targets for addressing bone-brain comorbidities based on these findings.

## 4 Impact of resistance exercise on skeletal muscles

### 4.1 The concept of resistance motion

Resistance exercise is any form of physical activity requiring muscle strength to counteract external resistance to increase muscle size, strength, and/or endurance (McArdle et al., 2015). Resistance exercise, or strength training, is a periodic form of physical exercise that uses external weight to overload and contract skeletal muscles, stimulating their strength and mass to lift more weight and increase muscle volume (Bodine et al., 2001). Resistance training includes exercises that increase muscle strength, endurance, and function by causing muscles to contract under external resistance. There are various types and forms of resistance exercises, including but not limited to bicep bending, overhead push, sitting rowing, squats, leg curling, knee extension, side hip elevation, and stretching exercise.

### 4.2 Impact of resistance exercise on AD

Over the past decade, RCTs have demonstrated that resistance training can improve cognitive function in healthy individuals and elderly people with cognitive impairments. Furthermore, recent studies indicate (Fonseca et al., 2025) that the effects of resistance training vary among distinct subgroups of cognitive impairment. Individuals exhibiting lower cognitive function demonstrate more pronounced adaptive responses in physical performance over time. The Excel for Cognition and Everyday Living study (Weier et al., 2012) is a 6-month randomized trial. Eighty-six elderly women aged 70–80 were randomly assigned to twice-weekly resistance, aerobic, and balance and tension training. It was observed that the resistance exercise group significantly improved their Stroop test and associative memory task abilities while also causing changes in the encoding and recall associations of three cortical regions (the fusiform gyrus of the right tongue and occipital region and the right frontal pole). In another RCT study on resistance training and cognitive training (Fiatarone Singh et al., 2014), patients with MCI were randomly assigned to two groups for 2–3 days/week of exercise for 6 months, followed up at 18 months and the AD Assessment Scale cognitive subscale (ADAS Cog), functional independence and Bayer Activities of Daily Living were observed. The ADAS Cog scores were significantly improved by resistance training.

In animal experiments, it has been observed that resistance exercise may improve cognitive impairment by promoting the clearance of Aβ in the hippocampus, reducing Aβ plaques and tau protein in the brain (Pena et al., 2020; Liu et al., 2020; Campos et al., 2023). AD is a neurodegenerative and neuroinflammatory disease characterized by the Aβ deposition, the formation of tau protein fiber tangles, excessive deposition of Aβ, and imbalance of inflammatory factors caused by neurotoxic activation of microglia. In the early stages of AD, microglia can promote the clearance of Aβ, but as the disease progresses, the neurotoxicity of microglia activates, causing them to lose their ability to clear Aβ. Microglia play a crucial function in the inflammatory factor signaling pathway in neurodegenerative diseases, including influencing the balance of pro-inflammatory [interleukin (IL)-1 β and tumor necrosis factor-alpha (TNF-α)] and anti-inflammatory factors (IL-4 and IL-10). A study suggests that moderate-intensity physical exercise, including resistance exercises, can suppress neurotoxic attacks by inhibiting the activation of microglia and reducing the expression of pro-inflammatory factors, including IL-1β and TNF-α (Mee-Inta et al., 2019; Spielman et al., 2016). Exercise can also increase the content of BDNF (López-Ortiz et al., 2021), reducing the levels of pro-inflammatory factors, including TNF-α, and alleviating cognitive impairment in AD. In 2007 research indicated that the expression of cytokines such as IL-4 and IL-13 was first identified in skeletal muscle following resistance exercise training. Furthermore, it was observed that the intensity of resistance exercise correlates positively with the levels of IL-4 in skeletal muscle (Prokopchuk et al., 2007). Subsequent investigations revealed that resistance exercise enhances the levels of anti-inflammatory cytokines, including IL-4 and IL-10, by modulating the activity of dendritic cells and microglia through the influence of TREM2, a transmembrane protein. This modulation facilitates a transition from a pro-inflammatory to an anti-inflammatory state, resulting in increased expression of IL-4 and IL-10, which may alleviate symptoms associated with AD (López-Ortiz et al., 2021; Forloni and Balducci, 2018; Leuchtmann et al., 2021). A recent study on the effects of resistance exercise on AD-related neurodegenerative diseases revealed that (Hashiguchi et al., 2020)resistance exercise reduces the volume of Aβ plaques in the hippocampus of APP/PS1 mice and maintains relatively stable levels of related cytokines in the hippocampus. Despite the absence of a significant decrease in Aβ protein levels under the combined action of anti-inflammatory and pro-inflammatory factors, the deposition of Aβ plaques was reduced, further improving cognitive impairment (Hashiguchi et al., 2020).

### 4.3 Effect of resistance exercise on skeletal muscles and the mechanism of improving cognition through the treatment of muscle atrophy

Resistance exercise improves cognitive function by reducing inflammation and enhancing muscle strength and physiological function. Multiple studies (McLeod et al., 2024; Vossel et al., 2024). have demonstrated that resistance exercise increases muscle mass, strength, and physical function compared to non-exercise. Enhancing muscle strength can lead to a greater release of irisin, which in turn raises the levels of IGF-1 and BDNF. This process helps reduce oxidative stress, encourages the growth of new neurons, and boosts insulin sensitivity (Someya et al., 2019). Moreover, resistance exercise can induce hypertrophy of skeletal muscle cells under the action of skeletal muscle satellite cells (Burns et al., 2010). In an animal experiment (Rahmati et al., 2023), 1–42 amyloid protein was injected in a single dose into the hippocampal Cornu Ammonis 1 region (1 μL/site). Rats with AD were compared with healthy rats after 5 weeks of resistance training. The findings indicated that AD induced significant skeletal muscle atrophy and reduced the number of muscle nuclei and muscle stem cell (SC) content in the gastrocnemius muscle in the entire muscle cross-section and isolated muscle fibers. Compared with the control group, there was no significant difference in the distribution of different myosin heavy chains (MyHC) in rats with AD, while resistance training significantly increased the muscle cross-sectional area of MyHC IIb fibers in AD and healthy animals. These results indicate that the skeletal muscles of AD animals are more prone to atrophy, and the number of muscle nuclei and satellite cell content are more easily lost, while resistance exercise can successfully restore these injuries. The increase in muscle nuclei and the recovery of satellite cells promoted muscle regeneration and anti-aging through related mechanisms (Sousa-Victor et al., 2022). This further indicates the role of resistance exercise in improving skeletal muscle physiological function and subsequently enhancing cognitive function. In an RCT study to determine whether improvements in aerobic capacity and strength after progressive resistance training (PRT) mediate cognitive function improvement, it was found that high-intensity PRT can significantly improve cognitive function in patients with MCI through increased muscle strength and aerobic capacity (Mavros et al., 2017). Moreover, a study on the effects of 12-week resistance training on the metabolism of the elderly brain found (Sheoran et al., 2023) that resistance exercise maintains the stability of various neurotransmitters to keep the brain at a relatively healthy level and significantly improves muscle strength. This increases the motor dependence of brain neurons to some extent, enhancing neural transmission and cognition. This may be because resistance exercise may promote the production and expression of myokines and angiogenic factors in large muscle groups (Fournier and Duman, 2012; Yeo et al., 2012), which cross the blood-brain barrier and promote long-term synaptic enhancement-induced signaling pathways, thereby enhancing the possibility of exercise-induced neuroplasticity (Vints et al., 2022) and improving brain function.

**Figure 1:**
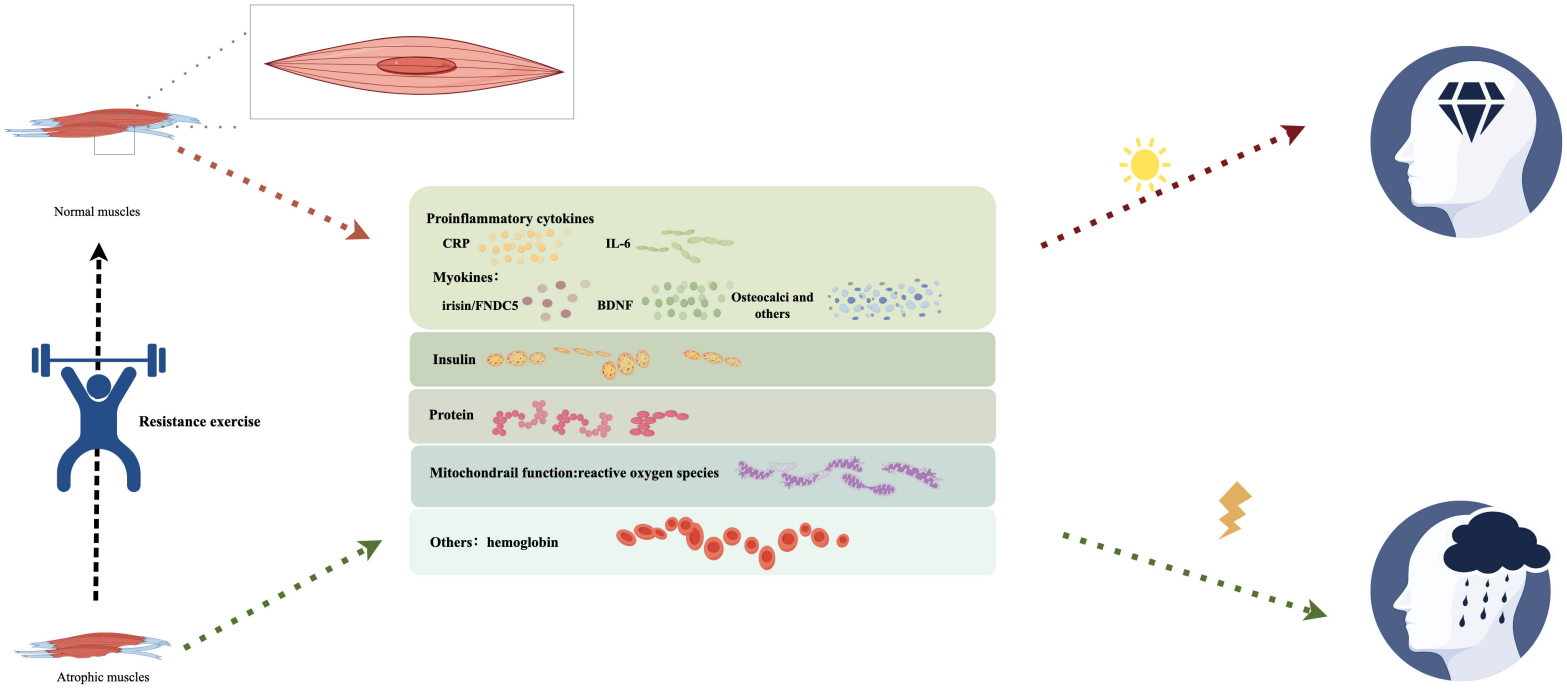
Resistance exercise, muscle atrophy and cognitive function.

Resistance exercise can induce oxidative stress and exert anti-inflammatory effects on cognitive function by increasing the release of neurotrophic factors, regulating inflammatory response, and reducing Aβ load in patients with AD (Navarro et al., 2018; de Almeida et al., 2022). Moreover, according to existing research, it directly affects skeletal muscle and cognitive function (Figure 1). Therefore, further research is required to elucidate the connection mechanism between resistance exercise, the physiological function of skeletal muscle, and the brain function of patients with cognitive impairment to further explore the potential of exercise in alleviating dementia, particularly in patients with skeletal muscle atrophy.

## 5 Best mode of resistance movement

In recent years, exercise has been extensively researched as a non-drug treatment for individuals with cognitive impairment. According to the American Physical Activity Guidelines (Piercy and Troiano, 2018), we categorized exercise interventions into four types: (1) aerobic exercise; (2) resistance exercise (RE); (3) multicomponent exercise (ME); and (4) mind-body exercise (MBE). A study by Bosserrs et al. in 2016 indicated (Bossers et al., 2016) that exercise can enhance daily living activities (ADL) in dementia patients, with a combination of aerobic and RE proving to be more effective than either type alone. A recent 12-week randomized controlled trial (RCT) conducted in 2023 highlighted (Papatsimpas et al., 2023) the benefits of resistance exercise in improving cognitive decline and instrumental activities of daily living (IADL) in dementia patients, particularly when paired with AE. This supports earlier findings that RE may offer greater advantages for cognitive function and knowledge-related outcomes (Li et al., 2018). This could be attributed to RE not only improving motor coordination and balance but also activating specific cerebellar-cortical connections, which may enhance both cognitive function and balance (Fonseca et al., 2025). A study investigating the impact of aerobic and RE on inflammatory factors and their relationship with neurocognitive performance found (Tsai et al., 2019) that after 16 weeks, both types of exercise positively influenced certain neurocognitive outcomes. The AE group experienced a notable increase in peripheral serum BDNF levels and a decrease in insulin, TNF-α, and IL-15 levels, while the RE group saw a significant rise in IGF-1 levels and a reduction in IL-15 levels. This suggests that in older adults with MCI, while both AE and RE can affect inflammatory factor levels and enhance neurocognition, the distinct inflammatory factors influenced by each type may indicate different molecular mechanisms at play in how they improve cognitive function.

The specific form, frequency, intensity, and duration of resistance exercise and the need to combine it with other non-pharmacological treatment methods to improve and delay the optimal combination mode of AD cognitive impairment remain the subjects of ongoing research. Aerobic exercise is more prevalent in daily life than resistance exercise because it can usually be completed without any specialized equipment, the movements are simpler, the exercise cost is lower, and it is more flexible. However, guidelines for patients with MCI recommend aerobic exercise and resistance exercise (Lautenschlager et al., 2018). This is because different forms of exercise have high specificity, and regular and appropriate resistance exercises also have unique effects on the skeletal muscles and cognition of patients with AD. The appropriate frequency, intensity, duration, and mode of resistance exercise are the manifestations of its specificity, which can more fully induce adaptation to the characteristics of the neuromuscular system and improve cognitive function.

### 5.1 Resistance exercise intensity

Research reveals that moderate-intensity resistance exercises achieve the greatest cognitive advantages (Chow et al., 2021). Moderate intensity training is defined as Chen et al. (2024) exercises that can be performed while maintaining uninterrupted dialogue, typically lasting 30–60 min (3–6 metabolic equivalents, 55–70% heart rate maximum, 40–60% heart rate recovery, 40–60% maximum oxygen consumption, the Rate of Perceived Exertion (PRE; C): 11–13, PRE (C-R): 3–4 [METs: metabolic equivalents; HRmax: heart rate maximum; HRR: heart rate recovery; VO_2_max: maximum oxygen consumption; Borg's RPE scales C=category scale (6–20) and C-R=category-ratio scale (0–10)]. This may be because moderate-intensity training optimally stimulates hormones, neurotransmitters, and other factors in the body through psychological and physiological factors, thereby promoting cognitive function. Low-intensity training is more conducive to physiological adaptation, whereas high-intensity induces fatigue and heightened wakefulness, reducing cooperation and completion for patients with cognitive impairment (Komiyama et al., 2020; Liu X. et al., 2024). A 2023 review report (Liu S. et al., 2024) examining the impact of acute exercise (both aerobic and resistance) on cognitive function in individuals with AD and MCI suggests that moderate-intensity acute aerobic and resistance exercise can improve inhibitory control (IC) in MCI patients. Conversely, high-intensity acute exercise does not appear to enhance IC, potentially due to the influence of BDNF and insulin-like growth factor 1 (IGF-1).

### 5.2 Duration of resistance exercise

Most trials last 12–52 weeks, with weekly exercise frequency varying from 1 to 3 times. Research reveals that regardless of the duration and frequency, regular resistance exercise can significantly improve the cognition of the elderly population. A preliminary study indicated that for female patients with MCI, resistance exercises were performed twice-weekly, with each group of exercises lasting 6–8 sessions for 6 months, and the cognitive abilities of this group of patients improved (Nagamatsu et al., 2012). This study was further supported by subsequent researchers, who conducted upper limb resistance training (2.4 kg dumbbells) and lower limb resistance training (chair test) separately on the patients, with 3 × 10 repetitions, 3 times weekly, for 12 weeks. The experimental group increased upper body strength by 58%, lower body strength by 68%, and cognitive ability by 19% (Smolarek Ade et al., 2016). In a recent study, participants aged 55 and older with MCI were subjected to resistance exercise for 6 months, 2–3 times a week. Physical and metabolic tests, a series of neuropsychological scale tests, and an MRI evaluation determined that regular resistance exercise can improve cognitive function and behavioral ability (Broadhouse et al., 2020). There was no significant statistical difference in the effectiveness of high-dose intervention (> 150 min/week) compared to low-dose intervention (< 150 min/week) in elderly individuals with cognitive impairment. Subsequently, certain scholars observed no significant correlation between cognitive function improvement and overall duration but suggested shorter and more frequent resistance exercises (Sanders et al., 2019). Although there is no clear requirement for the duration of resistance exercise in improving cognition, a minimum intervention time of ≥ 8 weeks is necessary to increase muscle strength and restore muscle function (Mayer et al., 2011). Moreover, skeletal muscles exhibit adaptability to resistance exercise; therefore (Liu S. et al., 2024), it is recommended that resistance exercise must be performed at least 2–3 times per week, lasting more than 12 weeks.

### 5.3 The form of resistance movement

The World Health Organization recommends that older adults must perform at least three major muscle exercises per week (Bull et al., 2020). A 12-week study comparing the effects of upper and lower-body resistance exercise on cognitive changes and physical function in older adults revealed (Sanchez-Lastra et al., 2022) that resistance exercise positively influences cognitive function and functional independence. Moreover, upper-body exercise was more effective for cognitive function, while lower-body exercise demonstrated superior improvements in physical function parameters. Resistance exercise training usually includes leg exercises, sitting rowing, chest exercises, latissimus dorsi pull-down, leg stretches, and triceps brachii flexion and extension (Timmons et al., 2018). For elderly patients with AD, the above movements can be optimized to avoid using heavy equipment and achieve the same objective by resisting their own weight. Each training session must be completed comfortably within 60 s while ensuring training safety and to ensure gradual overload throughout the entire training intervention process, thereby fully activating the function of skeletal muscles in resistance exercise.

We recommend resistance exercise programs targeting many major muscle groups throughout the body, with exercise frequency of 2 to 3 times a week, lasting over 12 weeks. Moderate-intensity training should be conducted, increasing weight gradually when each exercise's repetitions become easy. To optimize health outcomes, combine aerobic exercise with resistance exercise regularly.

## 6 Conclusion

In conclusion, restoring partial physiological structure and function of skeletal muscles can improve cognitive levels. Researchers have discovered that atrophied skeletal muscles can affect cognition through the secretion of hemoglobin. Conversely, resistance exercise can promote muscle secretion of related muscle factors and growth factors and directly improve skeletal muscle atrophy by increasing muscle strength, thereby reversing AD-related brain atrophy and alleviating cognitive dysfunction Consequently, resistance exercise can improve cognitive function and delay cognitive impairment in patients with AD by restoring skeletal muscle function. Currently, there is relatively little research regarding the causal relationship between AD and skeletal muscle atrophy, as most patients with late to scattered AD exhibit relatively late onset age, often accompanied by unavoidable complications, including osteoporosis and skeletal muscle atrophy. For the elderly, further research is essential to elucidate whether skeletal muscle atrophy affects cognitive function or if cognitive dysfunction exacerbates skeletal muscle atrophy. A precise comprehension of the relationship between the two can be achieved through targeted interventions to delay the disease's overall progression.

Among all treatment methods for AD, exercise is widely acknowledged and has a lesser economic burden on patients and their families. Accordingly, for patients with AD, especially those with skeletal muscle atrophy, resistance exercise can improve inflammation and brain function through vascular and nerve pathways and restore skeletal muscle physiological function, thereby achieving twice the result with half the effort in improving cognitive function. The optimal resistance exercise mode should involve as many major parts of the body as possible and may be integrated with aerobic exercise. Moderate-intensity exercise should be performed 2–3 times weekly for over 12 weeks. Currently, we recommend the most effective resistance exercise approach for all AD patients. Future research should explore whether tailored exercise regimens are necessary for individuals with varying levels of cognitive impairment, and if these can be integrated with other non-drug therapies to maximize clinical benefits for patients.
